# Nutrition in children with exocrine pancreatic insufficiency

**DOI:** 10.3389/fped.2023.943649

**Published:** 2023-05-05

**Authors:** Yuhua Zheng, Shikib Mostamand

**Affiliations:** ^1^ Gastroenterology, Hepatology and Nutrition, Children’s Hospital Los Angeles, Keck School of Medicine, University of Southern California, Los Angeles, CA, United States; ^2^ Gastroenterology, Hepatology, and Nutrition, Stanford Children’s Health & Stanford University School of Medicine, Palo Alto, CA, United States

**Keywords:** exocrine pancreatic insufficiency, pancreatitis, nutrition, fat-soluble vitamins, calories, malnutrition, PERT, cystic fibrosis

## Abstract

Exocrine pancreatic insufficiency (EPI) is a condition defined as pancreatic loss of exocrine function, including decreased digestive enzymes and bicarbonate secretion, which leads to maldigestion and malabsorption of nutrients. It is a common complication in many pancreatic disorders. If left undiagnosed, EPI can cause poor digestion of food, chronic diarrhea, severe malnutrition and related complications. Nutritional status and fat-soluble vitamins should be carefully assessed and monitored in patients with EPI. Early diagnosis of EPI is clinically important for appropriate nutritional support and initiating pancreatic enzyme replacement therapy (PERT) which could significantly improve patient outcomes. The evaluation of nutritional status and related unique management in children with EPI will be discussed in this review.

## 1. Introduction

The pancreas has two essential functions: exocrine function to help break down food by producing digestive enzymes and endocrine function to regulate blood sugar by secreting hormones including insulin. Exocrine pancreatic insufficiency (EPI) is a condition defined as pancreatic loss of exocrine function, including decreased digestive enzymes and bicarbonate secretion, which leads to maldigestion and malabsorption of nutrients. In pediatrics, more common causes of EPI include cystic fibrosis, chronic pancreatitis, Shwachman-Diamond syndrome, Pearson syndrome, and Johanson-Blizzard syndrome. Pancreatic hypoplasia, pancreatic aplasia, Jeune syndrome, pancreatectomy and isolated pancreatic enzyme deficiencies are less common causes (1–3). EPI can also occur in systemic diseases such as diabetes, inflammatory bowel disease, celiac disease, Sjogren's syndrome, etc. Microvascular damage may cause fibrosis and atrophy of the pancreas in patients with diabetes. Transient decrease of fecal elastase-1 has been reported in patients with inflammatory bowel disease or celiac disease (2) (Table 1). Clinically significant EPI presenting with steatorrhea results after greater than 90% of pancreatic acini are permanently compromised (3). If left undiagnosed, EPI can cause maldigestion of food and result in steatorrhea, weight loss, and fat-soluble micronutrient malabsorption. It can also impact the quality of life due to persistent gastrointestinal symptoms (4). To recognize EPI early is clinically critical for providing appropriate nutritional support including initiating pancreatic enzyme replacement therapy (PERT). In this review, we will discuss the evaluation of nutritional status and the management of EPI in children.

**Table 1:** Etiologies for EPI.

Pancreatic – focused diseases	Systemic diseases
Cystic fibrosis	Diabetes
Chronic pancreatitis	Inflammatory bowel disease
Shwachman-Diamond syndrome	Celiac disease
Pearson syndrome	Sjogren's syndrome
Johanson-Blizzard syndrome	
Pancreatic hypoplasia	
Pancreatic aplasia	
Isolated pancreatic enzyme deficiencies	
Jeune syndrome	
Pancreatectomy-partial or total including Total Pancreatectomy with Islet Auto Transplantation (TPIAT)	

## 2. Pathophysiology

The adult pancreas delivers approximately 2.5 L of fluid secretion to the duodenum daily. During meals, the flow can rise from 0.2 ml/min to 4.0 ml/min. In children, the secretion volume and rate in response to secretin has a strong correlation with body surface area (BSA) (5, 6). Pancreatic fluid is isotonic, slightly alkaline (pH∼8.2) and protein rich, containing HCO3 – (up to 140 mEq/L), other electrolytes, and water. Acinar cells synthesize, store, and release digestive proenzymes which are proteolytic, lipolytic, or amylolytic, and contain nuclease. These proenzymes are synthesized in the endoplasmic reticulum. Apices of acinar cells contain zymogen granules which are vesicles containing proenzymes. The release of zymogen into the intercalated ducts is controlled through receptors and mediated by calcium. These proenzymes are activated in the intestine following trypsin activation by enterokinase found on the mucosal surface of small intestine. Trypsin in turn activates the remaining proenzymes, including trypsinogen, by enzymatic cleavage. Ductal cells secrete 1–2 L of neutral pH juice, mainly water and HCO3–, devoid of Cl–, through the regulated action of the cystic fibrosis transmembrane conductance regulator (CFTR), intracellular carbonic anhydrase, and other membrane channels. Pancreatic secretion is regulated by hormones and neural mediators including secretin, acetylcholine (Ach), cholecystokinin (CCK), substance P, vasoactive intestinal polypeptide (VIP), Peptide YY (PYY) and gastrin-releasing peptide (GRP) (6–8).

Malabsorption usually occurs when pancreatic secretion is decreased by 90% or more (9, 10). In patients with chronic pancreatitis, steatorrhea represents the most significant digestive malfunction in EPI. It usually develops years before overt malabsorption of protein and starch and is often more severe than azotorrhea (11). This can be due to several mechanisms: (1) lipase secretion diminishes earlier compared to amylase and proteases (12); (2) lipase destruction in the small intestinal lumen occurs more rapidly than other enzymes (13); (3) lipid digestion in humans is almost entirely through pancreatic lipase. Lipolytic enzymes of gastric origin contribute little to lipid digestion. By contrast, if pancreatic proteolytic activity was inhibited, protein digestion was maintained in animal studies. Similarly, brush border oligosaccharidases and salivary amylase accomplish around 80% of starch digestion in the absence of pancreatic amylase (14); (4) ileal nutrient exposure variation caused by pancreatic exocrine insufficiency may impair ileal inhibitory effect and subsequently decrease biliary secretion (15). A decrease in bile acids may further worsen lipid digestion and absorption (11, 16, 13). Steatorrhea is often accompanied by diarrhea and enhanced gastric emptying and small intestinal transit in patients with EPI can contribute to this. Accelerated gastric emptying after a high-fat liquid meal was observed in patients with CP and EPI (17). These disorders can cause inadequate mixing of food, bile acid, and digestive enzymes, as well as reduce contact time between chyme and the intestinal mucosa leading to diarrhea (18). On the other hand, another study in patients with CF demonstrated a prolonged small intestinal transit time. This may increase the contact time of chyme with the mucosa, however, is also prone to the risk of developing intestinal bacterial overgrowth and contributing to secondary malabsorption (19).

HCO3- secretion is mediated through CFTR which drives osmotic fluid secretion in the pancreatic duct. HCO3- is required to control the pH at the epithelial surface of the pancreatic duct as well as the expansion of secreted mucins. In patients with CFTR mutation, a lower luminal pH could cause the accumulation of hyperviscous mucus in the pancreatic duct which subsequently obstructs the lumen and results in microbial colonization and inflammation of the pancreas (20, 21). Additionally, a high level of HCO3- is considered essential to maintain the inactive state of the secreted digestive enzymes while still located in the ductal tree (21, 22).

## 3. EPI and malnutrition

EPI causes malnutrition through maldigestion and malabsorption. Malnutrition is an imbalance between nutritional intake, basal energy requirements, and expenditure. Malnutrition can be further characterized as undernutrition or overnutrition. In this manuscript, we focus on undernutrition, thus, malnutrition in this review specifically refers to undernutrition. Pediatric undernutrition is defined as the state in which there is a deficit in nutritional intake in relation to requirements (23, 24). This results in cumulative macro or micronutrient insufficiencies or deficiencies which adversely affect growth and development (24).

### 3.1. Undernutrition

Patients with EPI often complain of abdominal discomfort, poor weight gain or weight loss, steatorrhea, undernutrition and vitamin deficiency symptoms (25). Steatorrhea is defined as bulky oily or greasy material in stools. Despite their often-late appearance in EPI, it remains important to evaluate the stool features even if these characteristics are neither specific nor sensitive for detection of steatorrhea (26).

Chronic malabsorption without intervention leads to malnutrition, more specifically undernutrition. One single center pediatric study identified malnutrition in 25% of children with chronic pancreatitis (CP). 17.3% of patients were detected with moderate malnutrition, and 0.96% with severe malnutrition. 152 patients were evaluated with CFA. The mean output among 38 malnourished patients was 6.69 g/100 g/day which was significantly higher than 2.27 g/100 g/day of 114 well-nourished children (27), indicating EPI is a major factor causing undernutrition. In patients with CF, protein malabsorption has been reported prior to newborn screen implementation, but is not common in the current era (28).

Other than maldigestion and malabsorption resulting from EPI, various factors may contribute to undernutrition owing to the nature of the underlying diseases. Patients with steatorrhea may self-limit fat intake due to diarrhea. Patients with chronic pancreatitis suffering from chronic pain may have decreased oral feeding. Those with additional complications such as diabetes, requiring frequent interventional procedures, having frequent pancreatitis attacks, or having a hypermetabolic state due to chronic inflammation such as cystic fibrosis leading to increased energy expenditure (29), may all potentially worsen or compound the undernutrition of patients with EPI.

Patients with steatorrhea are prone to develop fat-soluble vitamin deficiency. Because of the decrease of pancreatic enzymes, malabsorption and diarrhea, the deficiencies of water-soluble vitamins such as vitamin B12, folic acid, electrolytes such as calcium, magnesium, zinc, may also be detected (30). Vitamin B12 is bound to haptocorrin (HC) in the stomach; pancreatic proteases and pH changes degrades HC and transfer B12 to intrinsic factors in the duodenum for its absorption in the distal ileum. Despite the risks, the Vitamin B12 deficiency is still rare case in patients with EPI (31, 32). On top of changes in composition of fat and muscle tissue, undernutrition may result in homeostasis disruption on bone mass and mineral density (27).

In one adult study, 32 patients with chronic pancreatitis with exocrine pancreatic sufficiency (EPS) and 26 patients with EPI were measured for bone mineral density (BMD) and bone mineral content (BMC) using a dual-energy x-ray absorptiometry (DXA) method. The mean *z*-score of BMD was −1.16 ± 1.29 in EPS group and 1.32 ± 0.90 in EPI. For BMC, it was −1.02 ± 1.17 vs. −1.39 ± 0.987 respectively. In both groups mean 25 (OH)D and mean 1.25(OH)2D were below reference range. The author concluded that the patients with chronic pancreatitis and severe EPI, were at risk to develop significant bone loss (33, 34). Bone mineral content (BMC) and lean body mass (LBM) are more delicate indicators of undernutrition than BMI (36, 37). Computed Tomography (CT) and Magnetic Resonance Imaging (MRI) based methods to assess bone density and skeletal muscle mass are promising research fields to assess changes in body composition due to pancreas-related cachexia and osteopenia (37).

### 3.2. Fat-soluble vitamin deficiency

Vitamins are mainly acquired through diet as the human body cannot synthesize them on its own. There are nine water-soluble vitamins and four fat-soluble vitamins found in the diet. Fat-soluble vitamins are absorbed and stored in adipose tissues, liver, and muscle (38). Patients with EPI are prone to developing fat malabsorption, subsequently leading to insufficient absorption of fat-soluble vitamins (41).

Fat-soluble vitamins are divided further into subgroups according to their molecular structure. Vitamin A is classified into two forms: retinoids and carotenoid. Retinoids include retinol, retinal, and retinyl esters. Carotenoids, such as beta carotene, are plant sources (39, 41). Xerophthalmia and night blindness are associated with vitamin A deficiency (42, 43) along with decreased opacity of the cornea and dry conjunctiva. Vitamin A deficiency can damage the epithelial lining of the gastrointestinal, respiratory, and genitourinary tracts resulting from the dryness of epithelial cells, subsequently increasing risk of infection (38, 44).

The main forms of vitamin D are ergosterol (vitamin D2) and cholecalciferol (vitamin D3) (45). Vitamin D deficiency can lead to a disruption of bone mineralization, compromising the growth and strength of bones in children and affecting density of bones in adults, leading to rickets, osteomalacia, osteoporosis, and osteopenia (38). Vitamin D has also been discovered to have other vital functions besides bone health, such as, inhibiting cancer cell growth, assisting in infection control, and decreasing inflammation (46).

Tocopherols and the tocotrienols are vitamin E, each comprising of four subgroups (47). Vitamin E deficiency can lead to neurological problems, i.e., ataxia, dysarthria, lower limb areflexia, and peripheral neuropathy. In infants it is associated with hemolytic anemia.

Vitamin K is classed into the phylloquinones and menaquinones. The metabolism of each fat-soluble vitamin is complex, with lipid being essential for their absorption in the intestinal lumen and carrier proteins, or lipoproteins, required for transportation. The fat-soluble vitamins are conveyed to the adipose tissue, liver and muscle for usage and storage (48, 49, 38). Vitamin K deficiency can lead to coagulopathy, observed as subcutaneous bleeding with prolonged prothrombin time. Lack of vitamin K may also play a factor in poor bone density (38, 50).

## 4. Diagnosis of EPI

Early diagnosis of EPI in children remains challenging. Exocrine pancreatic function is often assessed by direct and indirect pancreatic function tests (PFTs). Indirect PFTs include fecal elastase-1 (FE-1), 72-hour fecal fat test, and triglyceride breath test mixed with 13^C^; the latter is not available in the United States (1, 3, 53, 55). Steatorrhea is traditionally diagnosed with 72-hour fecal fat test which measures the coefficient of fat absorption (CFA). Feces are collected for 3 days, and daily dietary fat intake is recorded. Fecal fat is measured. For patients over 6 months old, when stool fat excretion surpasses 7 g for every100 g fat taken from diet per day, i.e., unabsorbed stool fat is >7% of dietary fat, the patient has steatorrhea. If patient is less than 6 months old, >15% is diagnostic (10). The 72-hour fecal fat test is laborious and unpleasant for personnel to handle and is not commonly performed by most centers. FE-1 is currently the most widely used method to screen for EPI. In general, A FE-1 > 200 ug/g is considered normal, 100–200 ug/g is indeterminate and may associate with possible decreased exocrine pancreatic function, and a FE-1 < 100 ug/g is abnormal and most likely indicating EPI. FE-1 usually detects EPI in the severe range and may miss mild to moderate cases (1). The FE-1 level may also be affected if measured during diarrhea caused by other etiologies (non-steatorrhea) due to dilutional effect or during an acute pancreatitis episode when fewer digestive enzymes might be produced. The sensitivity for FE-1 in meta-analysis in mild EPI was reported around 49% (56). The lack of sensitivity and specificity of FE-1 in mild to moderate EPI limits its use as a reliable tool for early EPI detection. Dreiling tube test is a traditional direct PFT which involves the fluoroscopic placement of an oroduodenal tube, administration of secretin or CCK then intermittent suction through the tube to collect duodenal pancreatic secretion (53). This modality is cumbersome, uncomfortable, and time-consuming and is not generally performed in pediatric centers. Endoscopic pancreatic function test (ePFT), which directly measures the pancreatic exocrine function, is considered the most accurate and feasible modality in diagnosing EPI. An esophagogastroduodenoscopy (EGD) will be performed under general anesthesia. A secretin (0.2 mcg/kg) or CCK (0.04 mcg/kg) is administrated intravenously to stimulate the pancreas secretion, followed by suction of pancreatic juice from duodenum at the different time interval. Pancreatic digestive enzymes including amylase, lipase, trypsin and chymotrypsin as well as bicarbonate concentration would be measured at the various time interval (55). Currently the protocol used by each center varies. The North American Society for Pediatric Gastroenterology, Hepatology and Nutrition (NASPGHAN) Pancreas Committee published a position paper that reviewed the advantages of ePFT, including it being technically safe and easy to perform, although it should be recognized that it is still an invasive procedure and carries risks of standard anesthesia and EGD. EPFT modality has been proved to be sensitive and specific in EPI diagnosis. The NASPGHAN pancreas committee proposed a standard ePFT protocol in children (56). Compared to the Dreiling tube pancreatic function test, ePFT is the preferred direct pancreatic function test given its technical superiority with improved efficacy. Despite its advantages as the most promising method for early EPI diagnosis, the main limitation for ePFT in children is the lack of age-specific normal reference ranges, often making it difficult to reliably interpret the results (56). Secretin-enhanced magnetic resonance cholangiopancreatography (sMRCP) is often utilized to investigate the pancreatic and biliary ductal system. Secretin-enhanced secretion of pancreatic fluid may contribute to non-invasive diagnosis for EPI. Calculation of pancreatic secretory function to secretin stimulation by MRI has been evaluated for adult patients and still being investigated in children (5).

## 5. Monitoring nutritional status and management in EPI

### 5.1. Overall malnutrition assessment

Appropriate assessment of nutritional status is essential for early identification of those at risk for malnutrition with EPI. There is a current lack of a standard, validated, universal approach to the screening and evaluation of pediatric malnutrition. Routine assessment remains inconsistent both nationally and internationally. The American Society for Parenteral and Enteral Nutrition (ASPEN) and Academy of Nutrition and Dietetics Consensus guidelines recommend using several indicators in evaluating malnourishment: caloric and nutrient intake, calculation of energy and protein needs, and physical exam findings (i.e., muscle wasting, subcutaneous fat thickness, fluid accumulation, Tanner staging). Diagnostic parameters include anthropometric measures and their proxies such as weight gain velocity, mid-upper arm circumference, growth parameters, and handgrip strength (23). In the United States, it has become standard practice to use the World Health Organization Multicenter Growth Reference Study for children less than 2 years of age (https://www.who.int/childgrowth/standards/weight_for_height/en/), and the Centers for Disease Control and Prevention growth charts as references for children greater than 2 years of age (https://www.cdc.gov/growthcharts/clinical_charts.htm) (57).

Current recommendations support the use of “*z*-scores” in the evaluation of pediatric nutrition. Utilizing *z*-score criterion is especially useful when a single data point is available such as weight for height or length (0–2 years), BMI for age (2–18 years), length or height for age, or middle upper arm circumference (MUAC) (23). The *z*-score is a statistical representation of the number of standard deviations (SD) which is a value either above or below the mean in a normal Gaussian (bell) curve distribution (57). One standard deviation from the mean encompasses 68% of the data set under the bell curve, 2 standard deviations from the mean encompasses 95% of the data, and 3 standard deviations from the mean encompasses 99.7% of the data. Thus, the *z*-score implies variance from a normal mean value, and more specifically – the degree of variation. With z-scores, pediatric undernutrition can be classified by severity. Mild malnutrition is specified as a *z*-score −1 to −1.99, moderate malnutrition is specified as a *z*-score −2 to −2.99 and severe malnutrition is a *z*-score equal to or less than −3 (23) (Table 2).

**Table 2:** Assessment of malnutrition.

Grade	Percentage of TBW lost	Growth velocity	Inadequate nutrient intake	*Z*-scores (single value)	*Z*-scores (multiple values)
Mild	5%	<75% of normal	51%–75% estimated caloric/protein need	−1 to −1.99	Decline of 1 *z*-score
Moderate	7.5%	<50% of normal	26%–51% estimated caloric/protein need	−2 to −2.99	Decline of 2 *z*-scores
Severe	10%	<25% of normal	<25% estimated caloric/protein need	≤−3	Decline of 3 *z*-scores

TBW, total body weight.

*Z*-score criterion is for anthropometric measures i.e., weight for height or length (<2 yo), BMI for age (2–18 yo), length or height for age, or middle upper arm circumference (MUAC). *Z*-score criterion is especially useful when only a single data point is available.

When multiple data points are available for use, *z*-scores for deceleration in weight for length or height may be used, in which case, a decline of 1 *z*-score correlates with mild malnutrition, a decline of 2 *z*-scores correlates with moderate malnutrition and a decline of 3 *z*-scores correlates with severe malnutrition (23) (Table. 2).

The nutrition status of patients with EPI requires close monitoring and documentation at each visit.

### 5.2. PERT

Pancreatic enzyme replacement therapy (PERT) can often reverse the clinical course of malabsorption. PERT is required for EPI patients to support weight gain, to prevent fat-soluble vitamin and essential fatty acid deficiencies, to avoid malnutrition, as well as to improve symptoms of maldigestion and steatorrhea (58). 80%–90% of patients with cystic fibrosis need PERT to avert malnutrition (59). Current understanding of PERT in EPI is mainly based on expert consensus experience from cystic fibrosis.

For PERT, porcine pancreas is the usual source of pancreatic enzymes. It features high enzyme activity of all three classes including amylases, lipases, and proteases. Lipase is the main supplemental pancreatic enzyme yet is the least stable. It is highly sensitive to acid environment and proteolysis (60). Since 2010, several pancreatic enzyme replacement products were approved for the treatment of EPI by the Food and Drug Administration (FDA). All brands but VIOKACE are available in delayed-release forms comprising enteric-coated spheres, microspheres, microtablets or beads. Enteric-coated enzymes safeguard lipase from denaturation caused by gastric acid. These products include Creon, PANCREAZE, ZENPEP, PERTZYE (61). VIOKACE is the only one with an uncoated enzyme formulation. It has an immediate release thus it should be used together with an acid suppressant medication i.e., proton pump inhibitor (PPI) to maximize its activity (2, 62). In general, delayed-release (enteric-coated) capsules are recommended for pediatric patients. The safety and effectiveness of VIOKACE has not been established in pediatric patients. Due to greater degradation in the acidic environment, VIOKACE may be less efficacious than enteric-coated formulations (63, 64). VIOKACE used alone in pediatric patients may increase the risk of inadequate treatment of EPI. The efficacy of VIOKACE was established with concomitant PPI therapy in adult patients (63, 64). The long-term safety of PPI use in pediatric patients has not been established. RELiZORB is a digestive enzyme cartridge that connects directly to the feeding tube. The enzyme lipase is attached to small bead carriers and interacts with lipid as the formula passes through. It is useful for patients who are tube fed, however, it only contains lipase to breakdown fat in the formula (65).

Once ingested and passed through the duodenum, the acid-resistant enteric coating degrades in the intestine's higher pH, permitting the release of enzymes for digestion. PERT should be taken with meals and snacks (2, 66).

The goal of PERT is to optimize the nutritional status and alleviate the symptoms. The optimal dosage for an individual may differ based on body weight, severity of steatorrhea, and dietary fat intake. In pediatrics, PERT dosing recommendations for EPI is age dependent. For infants, 2,000–4,000 lipase units (LU) per 120 ml of infant formula or each breast feeding is recommended. For under 4 years of age, 1,000 LU/kg/meal and 500 LU/kg/snacks are recommended. For greater than 4 years of age, 500 LU/kg/meal and 250 LU/kg/snack are recommended (3) (Table 3). The ideal PERT therapy is established on its clinical effectiveness, the initial dose might be adjusted based on the clinical requirement and efficacy (2, 34, 67–69).

**Table 3:** PERT dosage by Age.

Age	PERT dosage in Lipase Units (LU)
Infant	2,000–4,000 LU per 120 ml of infant formula or each breast feeding
<4-year-old	1,000 LU/kg/meal and 500 LU/kg/snacks
>4-year-old	500 LU/kg/meal and 250 LU/kg/snack

PERT, pancreatic enzyme replacement therapy.

**Table 4:** Fat soluble vitamins supplement.

Vitamins	Dosage
**Vitamin A**	
Birth to 6 months	400 mcg of (RAE)
Infants between 7 and 12 months	500 mcg RAE
1–3 years	300 mcg RAE
4–8 years	400 mcg
9–13 years	600 mcg RAE
Boys at 14–18 years	900 mcg
Girls at14–18 years	700 mcg RAE
**Vitamin D**	
Infants	400–500 IU daily
1–10 years	800–1,000 IU daily
>10 years	800–2,000 IU daily
Vitamin D deficiency without hypoglycemia	Vitamin D ranging from 25 to 125 mcg (1,000–10,000 IU) per day should be provided for 8–12 weeks to quickly correct the deficiency, then continue 10–25 mcg (400–1,000 IU) per day as maintenance.
Vitamin D Deficiency with hypocalcemia	Additional 30–75 mg/kg/day of elemental calcium
**Vitamin E**	
Infants	40–50 IU
toddlers	80–150 IU
4–8 years	100–200 IU
>8 years	200–400 IU
Vitamin E Deficiency	start at 10–25 IU/kg/day and may be increased by small increments (25–50 IU/kg/day every 3–4 weeks) to a maximum of 100–200 IU/kg/day, best offered as a single morning dose
**Vitamin K (with prolonged INR)**	
	2.5–5 mg oral or 1–2 mg I.M., I.V., subcutaneous

RAE, retinol activity equivalents.

For children with difficulty swallowing capsules, delayed release forms may be opened, and enteric coated microspheres may be sprinkled on low pH food (applesauce, etc). Foods like milk has a pH greater than 7.3, should be avoided as the enteric coating may dissolve in higher pH, and the enzymes can be denatured by gastric acid and lose activity. It is recommended to avoid crushing or chewing or holding the pancreatic lipase in the mouth as this may cause local irritation (3, 70). As the dissolution rate and extent of each brand are unique, they are not deemed interchangeable (2). If a different brand of PERT is initiated, optimization of the new PERT dose should be considered.

PERT products are generally well tolerated. Over time, it has shown an acceptable safety and tolerability profile. Headache, dizziness, abdominal pain, gassiness, and diarrhea are commonly observed adverse effects (71). In cystic fibrosis patients, fibrosing colonopathy has also been described with higher doses (72). According to 1995 consensus conference of the U.S. Cystic Fibrosis Foundation on the use of PERT, it is recommended that the daily dose of pancreatic enzymes should not exceed 2,500 LU per kilogram per meal and 10,000 LU per kilogram per day. Higher doses should be used with vigilance and only if able to clinically demonstrate significant improvement of malabsorption (2, 72, 73).

Regardless of the causes of EPI, a suboptimal response to standard PERT dosage should lead clinicians to investigate adherence to therapy first. If adherence is satisfactory, a small increments of PERT dosage change is recommended. Acid suppressive therapy (i.e., PPI) to reduce acid denaturation of enzymes can be initiated (74, 75). It has been suggested that combining a proton pump inhibitor (PPI) in cystic fibrosis patients who have refractory steatorrhea not responding well to PERT will aid efficacy (34, 67–69). However, in a retrospective cohort of pediatric patients with cystic fibrosis treated with PERT jointly with PPIs, there was no statistically significant improvement (76). In an adult study, up to 40% of patients with EPI secondary to chronic pancreatitis have concomitant intestinal bacterial overgrowth (77). Alternative etiologies of malabsorption should be evaluated in cases of less ideal response to the treatment (2, 74, 75).

### 5.3. Fat-soluble vitamins

Children with EPI are prone to develop fat-soluble vitamin deficiency. The Cystic Fibrosis Foundation (CFF) recommends regular screening for deficiencies in fat-soluble vitamins at the time of diagnosis and then annually and after any dose change (78) And children with CP should have fat-soluble–vitamin levels measured every 6–12 months (79, 80). If patients are supplemented with vitamins, levels should be monitored 3 months after dose adjustment (80). Fat-soluble vitamins deficiency is secondary to fat malabsorption, improvement is expected with optimized PERT supplement. Fat-soluble vitamins should be supplemented for deficiencies accordingly.

#### 5.3.1. Vitamin A

The predominant circulating vitamin A is in the form of retinol. Serum retinol levels are not useful in assessing vitamin A body stores. They reflect vitamin A storage in the liver when they are either depleted (less than 0.07 µmol/g liver) or exceedingly high (greater than 1.05 µmol/g liver) (51). In the middle of these levels, serum retinol is physiologically well controlled and kept at a homeostatic range. Thus, its level is not correlated with vitamin A deficiency and may not correlate in response to vitamin A supplementation. The serum retinol is useful when measured in a population and provides valuable information on the vitamin A status of a population. The serum retinol defines whether vitamin A deficiency is a public health problem in that population (56, 85). Vitamin A is bound to retinol-binding protein (RBP) for transportation. RBP is produced in liver. The molar ratio of retinol to RBP can be assessed to guide if vitamin A supplementation is necessary in patients with malnutrition or liver disease. A ratio of <0.8 suggests true vitamin A deficiency and requirement of vitamin A supplementation (81, 78).

Vitamin A is found in fruits, vegetables, eggs, milk, meat, and seafood. The daily Recommended Dietary Allowance (RDA) for vitamin A in children is age dependent (Table 4). According to National Institutes of Health (NIH): During birth to 6 months, 400 mcg of retinol activity equivalents (RAE) is recommended, Infants between 7 and 12 months require 500 mcg RAE; Children at 1–3 years require 300 mcg RAE; Children at 4–8 years require 400 mcg RAE; Children at 9–13 years 600 mcg RAE; teenage boys at 14–18 years require 900 mcg RAE; and teenage girls at14–18 years require 700 mcg RAE. This intake level is easy to reach if plenty of whole foods are consumed. However, to prevent toxicity, it is important not to exceed the 3,000 mcg per day (83). There are two supplements form of vitamin A are available: provitamin A as carotenoids and pre-formed vitamin A as retinol or retinyl ester. If a product contains both, the amount of pre-formed vitamin A is used to determine if it is safe. It is important to note that vitamin A may also be an ingredient in some topical products, such as serums, creams, and lotions (83). Toxicity of hypervitaminosis A secondary to the supplement is rare, it may involve multiple organ system including the bone, nervous system, kidney and liver (84). Hypercalcemia due to vitamin A toxicity was reported in patients with CF (85).

#### 5.3.2. Vitamin D

Vitamin D level can be classified as severe deficiency (<5 ng/ml), deficiency (5–15 ng/ml), insufficiency (15–20 ng/ml), and sufficiency (20–100 ng/ml) (86). In children, 20 ng/ml for 25(OH)-D levels is still considered sufficiency (87, 88), however, a higher cutoff of 32–100 ng/ml is suggested in adults (88). The US Cystic Fibrosis Foundation recommends levels >30 ng (89).

Vitamin D is naturally present in some foods, fatty fish and fish liver oils are some the best sources (90). It is also produced endogenously when skin is exposed to ultraviolet (UV). In foods and dietary supplements, D2 (ergocalciferol) and D3 (cholecalciferol) are the two main forms, which differ only in their sidechain (90, 91). UV light converts cutaneous 7-dehydrocholesterol to previtamin D3, which subsequently transform into vitamin D3 (90, 92). Pharmacologic doses of vitamin D ranging from 25 to 125 mcg (1,000–10,000 IU) per day should be provided for 8–12 weeks to quickly correct the deficiency, and once corrected, then 10–25 mcg (400–1,000 IU) per day should be continued as maintenance. Patients with hypocalcemia may need calcium supplementation, in which case, 30–75 mg/kg/day of elemental calcium should be offered. It is recommended to start at a higher dose then wean down to the lower range (Table 4). Vitamin D level should be monitored during the therapy (86). Toxicity of hypervitaminosis D is also rare which may be related to excessive long-term vitamin D intake. Clinically characterized by symptoms associated with severe hypercalcemia (93).

#### 5.3.3. Vitamin E

Alpha and gamma tocopherol levels are monitored in laboratory. Alpha Vitamin E reflects vitamin E mainly from supplement and gamma Vitamin E from food intake primarily from plant.

For vitamin E deficiency, oral vitamin E is available in standard forms of tocopherol, tocopherol acetate, tocopherol succinate or tocopherol nicotinate. The dose is recommended to start at 10–25 IU/kg/day and may be increased by small increments (25–50 IU/kg/day every 3–4 weeks) to a maximum of 100–200 IU/kg/day (Table 4). Since bile flow is maximal with breakfast in the morning, vitamin E is best offered as a single morning dose (81, 97).

#### 5.3.4. Vitamin K

Serum vitamin K level is not very useful in reflecting the deficiency status, as it only indicates the vitamin K intake over past 24 h (98). Protein induced by vitamin K absence or antagonism (PIVKA) and des-gamma-carboxy-prothrombin (PIVKA-II) are functionally defective coagulation factors in vitamin K deficiency status, PIVKA-II and undercarboxylated osteocalcin (uc-OC) are sensitive markers to reflect vitamin K deficiency but are not available at clinical settings (99–101). In clinical practice, prothrombin time/International Normalized Ratio (PT/INR) is usually used to reflect vitamin K deficiency (99). However, INR prolongs when prothrombin level is below 50% of normal, thus it does not identify early vitamin K deficiency (95). PT/INR measurement is indirect, less sensitive but more readily available in clinical settings.

Phylloquinone is the main dietary form of vitamin K coming primarily from green leafy vegetables (100, 101). Menaquinones are mainly of bacterial origin from various animal-based and fermented foods (100, 102, 103).

If prolonged INR is considered secondary to vitamin K deficiency, 2.5–5 mg oral or 1–2 mg I.M., I.V., subcutaneous (SC) vitamin K1 could be given as a single dose (104) (Table 4). The PT/INR could be normalized as soon as within 30 min after intake (105). Cystic fibrosis foundation recommended for patients with EPI, oral 0.3–0.5 mg/day high doses vitamin K1 may be administered until PERT supplement is optimized (106). If oral dosing is ineffective, alternative route of vitamin K1, i.e., IM/IV/SC should be considered (107).

### 5.4. Other nutrients

#### 5.4.1. Water-soluble vitamins, trace elements and minerals

Research data in studying water-soluble vitamin deficiencies in pediatric patients with EPI is limited. Vitamin C and vitamin B12 level could be low in adults patients with CP (80, 108, 109).

Patient with severe phenotype of CF often has EPI, who may experience iron deficiency due to GI tract or sputum loss (110). Patients with CF have abnormal transport of sodium and chloride in the sweat glands. Sodium chloride deficits can be problematic in infant with CF (99). Selenium levels are low in patients with CP or CF (113, 114). In patients with inadequately treated EPI, zinc deficiency can occur due to steatorrhea (113). Calcium requirements should be optimized in patients with EPI which is important for bone health along with Vitamin D and Vitamin K (114). Specific dosage recommendations for water-soluble vitamin supplementation do not exist in CF (99). Unless suspecting deficiencies, routine screening of water-soluble vitamins, trace elements or minerals are not recommended in pediatric patients with chronic pancreatitis (80).

#### 5.4.2. Essential fatty acid

Essential fatty acid deficiency (EFAD) can be observed in patients with CF (99). Essential fatty acids include polyunsaturated fatty acids which can be metabolized to alpha-linolenic acid (n-3) and linoleic (n-6). N-3 fatty acid is metabolized to docosohexaenoic acid (DHA) and N-6 fatty acid is metabolized to arachidonic acid (AA). The benefits of supplementation of antioxidants or DHA were observed in some studies; however, they were not consistent to recommend routine supplements yet (99, 116).

### 5.5. Other support

A balanced diet with carbohydrates, protein, fat, vegetables and fruits should be encouraged. The American Heart Association recommends that 30%–35% of calories are derived from fat for children 2–3 years of age and 25%–35% for 4–18 years of age. The majority of fats should come from polyunsaturated and monounsaturated fatty acid sources like fish, nuts and vegetable oils (117). In patients with a hypermetabolic state due to chronic inflammation (i.e., cystic fibrosis), it is important to achieve the recommended high-energy level intake at 110%–200% of the estimated average requirement (EAR) given increased energy expenditure (79, 118). It is also important to encourage children to remain well hydrated and refrain from consuming alcohol or tobacco products which are associated with CP and subsequent EPI (118). The mechanisms underlying malnutrition in patients with EPI can be complex. Like patients with CF, factors such as higher energy demands, greater energy losses, decreased nutrient intake, and declining lung function all contribute to poor nutrition status and necessitate special attention. Ideally, a multidisciplinary team including registered dietitians, pharmacists, registered nurses, clinicians and social workers can be of great help to manage individual nutritional and caloric requirements.

Other than maldigestion and malabsorption secondary to EPI, various factors may contribute to undernutrition owing to the nature of the underlying diseases. Specific measures for the underlying disease should be considered. For patients with steatorrhea who may self-limit fat intake due to diarrhea, it is important to adjust PERT dosage and encourage a balanced diet. Optimizing pain control for patients with chronic pancreatitis and chronic pain is also important.

## 6. Summary

Exocrine pancreatic insufficiency (EPI) is a common condition in patients with pancreas disorders which leads to maldigestion and malabsorption of nutrients. Early diagnosis of EPI is clinically important for appropriate nutritional support and initiation of PERT. The nutritional status of patients with EPI should be assessed carefully and accurately. The goal of PERT is to optimize the nutritional status and alleviate symptoms. Fat-soluble vitamins deficiency is secondary to fat malabsorption, improvement is expected with optimized PERT supplement. Fat-soluble vitamins should be supplemented for deficiencies accordingly. Additional support may be needed to improve patient outcomes.
